# Cervicomastoidfacial versus modified rhytidectomy incision for benign parotid tumors

**DOI:** 10.5935/1808-8694.20130030

**Published:** 2015-11-02

**Authors:** Agnaldo José Graciano, Carlos Takahiro Chone, Carlos Augusto Fischer

**Affiliations:** a MSc in Otorhinolaryngology and Head and Neck Surgery - Federal University of São Paulo (UNIFESP) - Paulista School of Medicine EMP). Physician in charge of the Department of Otorhinolaryngology and Head and Neck Surgery - São José Hospital - Joinville/SC); b PhD, Professor - Department of Otorhinolaryngology and Head and Neck Surgery - State University of Campinas (UNICAMP); c MD. Head and Neck Surgeon and Maxillofacial Surgeon - Head and Neck Surgery Department - São José Hospital - Joinville SC. Otorhinolaryngology and Head and Neck Department - Hospital São José

**Keywords:** otorhinolaryngologic surgical procedures, parotid gland, parotid neoplasms, parotid region

## Abstract

The modified rhytidectomy incision is an alternative to the classic cervicomastoidfacial approach for parotid surgery, camouflaging the scar in barely visible areas, resulting in better cosmesis. However, there are very few studies comparing the incidence of complications and functional results of patients submitted to parotidectomy through these two different approaches.

**Objective:**

Compare the incidence of complications and functional results of patients with benign parotid neoplasms submitted to surgery through the classical incision versus the modified rhytidectomy approach.

**Method:**

Retrospective cohort study evaluating the demographics, surgical and post-operative characteristics of an equally distributed group of sixty patients submitted to parotidectomy via cervicomastoidfacial incision or modified rhytidectomy approach.

**Results:**

There were no significant differences in complications rates and functional results between the groups, except for a lower incidence of early facial movement dysfunction for the modified rhytidectomy approach - which was 86% lower in this group of patients.

**Conclusion:**

Modified rhytidectomy incision has shown comparable complication rates to those of the classic approach and a lower incidence of immediate facial movement impairment.

## INTRODUCTION

The modified rhytidectomy (MR) approach for parotid surgery was proposed as an alternative to the classical cervicomastoidfacial incision (CMFI), to avoid a scar in the anterior neck region, which can be perceived in some patients and it is not well accepted by younger individuals^1^. Some authors started to prefer this modification to operate benign tumors, especially for the possibility of keeping the incision hidden in the hairline, with a better cosmetic result as far as the scar is concerned, but without compromising surgical exposure and increasing surgery time^2^, ^3^, ^4^, ^5^. Recent studies have suggested that such approach is also associated with a lower degree of facial movement dysfunction^6^. Notwithstanding, there is very little comparative data on the functional results obtained from patients submitted to parotidectomy by these different approaches.

Therefore, we aimed at comparing clinical, surgical and functional aspects from patients submitted to parotidectomy and treated by the modified rhytidectomy or CMFI.

## METHOD

This is a longitudinal historical cohort, assessing 95 patients submitted to parotidectomy in a specialized service between March of 2004 and March of 2010, and approved by the Ethics in Research Committee of our Institution. We included 60 patients submitted to partial parotidectomy and with a pre-operative clinical and/or cytological diagnosis of benign neoplasia of the parotid gland, followed by at least 6 months after the treatment, with an equal distribution of patients between the groups submitted to MR (group A = 30) or CMFI (group B = 30).

As for exclusion criteria, we considered the cases suspected of malignancy with a plan for total parotidectomy, associated or not to neck dissection, and patients with chronic sialadenitis. The variables of interest analyzed were: age, gender, hospital stay, volume of secretion drained in the post-op, tumor size and volume of the resected parotid, salivary fistula and facial movement dysfunction - characterized by the limitation of facial muscle groups detected by the physical exam and considered immediate if observed up to the first week after surgery, and late if present up to six months after the procedure.

### Surgical approach

All the patients were submitted to venous and inhalation general anesthesia and the surgeries were carried out without the use of intraoperative monitoring of the facial nerve. We positioned the patient in dorsal decubi-tus with neck extension and lateralization, prepared the operatory field and injected saline solution with 1:100.000 UI of adrenaline in the cervicofacial region to achieve a proper level of vasoconstriction. For the classical CMFI approach, one incision in the shape of a bayonet is made anteriorly to the ear contour with a slight curvature on the lobule line, followed by anterior neck extension, at 3 cm away from the mandible angle, and the fasciocutaneous flap is raised at the superficial aponeurotic muscle system (SAMS) plane with gland exposure, as shown by others^7^.

**Figure 1:**
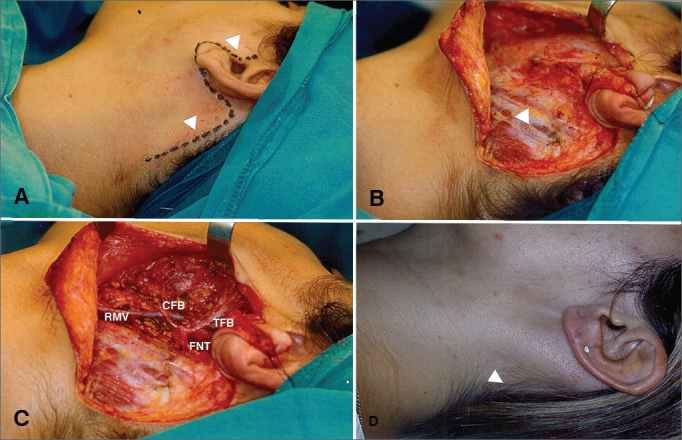
Superficial parotidectomy via modified rhytidectomy. A: Drawing of the pre-auricular incision extending to the hairline (arrows). B: Dissection of the fasciocutaneous fap; exposure of the parotid and anterior border of the sternocleidomastoid muscle (arrow). C: Surgical field view after resection of the parotid's superficial portion (FNT: Facial nerve trunk; CFB: Cervicofacial branch; TFB: Temporofacial branch; RMV - Retromandibular vein). D: Late scar hidden in the hairline (arrow).

Concerning the access with the MR, the anterior incision in the ear contour is posteriorly continued on to the ear lobule fold, and following the behind-the-year incisure all the way to the hairline, where it follows by an inferior curvature for about 5 to 10 cm. We then raise the SAMS fasciocutaneous flap for parotid exposure in order to locate the facial nerve and resect the gland's superficial portion (Figure 1). The same reference points are used in both surgical approaches, which are the anterior border of the sternocleidomastoid muscle at the tip of the mastoid, the posterior belly of the digastric muscle and the cartilaginous tip of the tragus, enabling the identification of the facial nerve trunk and the careful dissection of its branches in order to raise the superficial portion of the gland en block together with the tumor.

### Statistical analysis

The categorical variables were shown in a descriptive fashion, with counts and proportions. Proportion comparison between the two independent groups was established with the chi-squared test or Fisher's exact test -when adequate. Mean values from quantitative variables were compared between the two independent groups by using the *Student-t test*. The assumptions of variance equality and normality from such tests were assessed using the Shapiro-Wilks and F test, respectively. When the *t-test* assumptions did not materialize, we used the Wilcoxon non-parametric test. The variables compared by the *t-test* and the Wilcoxon test were described as mean (±standard deviation) and median (interquartile interval), respectively. All significance probabilities (*p* values) shown were bilateral and values below 0.05 were deemed statistically significant. The results were also submitted to the exact multivariate logistic regression analysis. The statistical data analysis was carried out using the SAS version 9.2 (Statistical Analysis System, Cary, NC, USA).

## RESULTS

**Graph 1:**
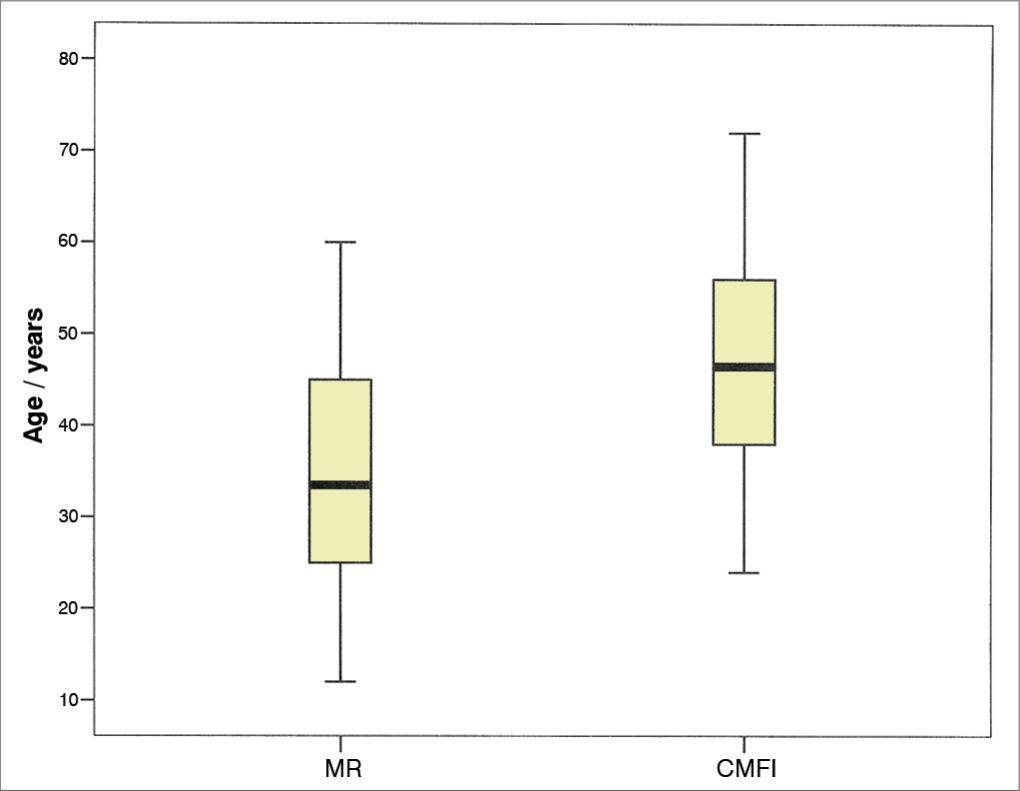
Disorders operated. Percentage distribution.

**Graph 2:**
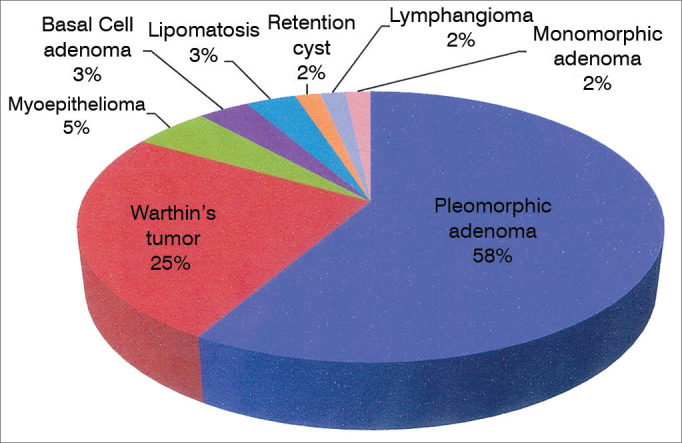
Age distribution among the groups. Mean age MR (34.93) and CMFI (47.30), *p* = 0.0003.

We assessed 60 patients with benign parotid tumors (Graph 1), 32 men (53.4%) and 28 women, distributed in two groups according to the type of incision made. Significant differences were seen for demographic variables of age (Graph 2) and gender distribution (Table 1), as well as for the surgical variables of total volume of resected parotid (mean 34.29 cm^3^ in Group A and 48.12 cm^3^ in Group B, *p* = 0.0428), immediate (23.33%) in Group A versus 73.33% in Group B, *p* = 0.0001) and late (10% in Group A 36.7% in Group B, *p* = 0.0146) facial movement dysfunction. The other variables: hospital stay duration, post-operative drainage volume, resected tumor size and post-operative salivary fistula were not significantly different between the two groups (Tables 2 and 3).

**Table 1:** Gender distribution between the groups.

Incision	Gender		Total
	M	F	
N	11	19	30
MR	34.4%	67.9%	
N	21	9	30
CMFI	65.6%	32.1%	
Total	32	28	60

N: Number of individuals. Higher N of women operated via MR (*p* = 0.01).

**Table 2:** Comparing the mean values of surgical variables.

	MR		CMFI		*p*-value
	Min-max	Mean	Min-max	Mean	
Hosp. stay/days	1-8	2	1-5	2	0.262
Drainage/ml	0-425	39	0-220	30	0.328
Tumor/cm	0.9-5.3	2.34	0.5-6.1	2.58	0.358
Parotid volume/cm^3^	9.45-115.42	34.29	12-166	48.12	0.0428

**Table 3:** Incidence of complications between the groups.

Complication	MR	CMFI	*p*-value
IFMD	23.33%	73.33%	0.0001
LFMD	10%	36.70%	0.0146
SF	13.30%	6.7%	0.102

IFMD: Immediate facial movement dysfunction; LFMD: Late facial movement dysfunction; SF: Salivary fistula.

The multivariable analysis showed that the likelihood of immediate post-operative facial movement dysfunction was 86% lower for individuals of the group submitted to the MR approach, when compared to Group B individuals (CMFI) after age-adjustment, Adjusted OR (exact) = 0.142, CI95% (exact) 0.032-0.539, *p* (exact) = 0.0022; and the same was not observed for the late motor dysfunction, the age-corrected multivariate analysis did not show differences between the two groups (adjusted OR = 0.263, CI95% 0.039-1.293, *p* = 0.1172).

## DISCUSSION

Partial parotidectomy is the treatment of choice for most benign parotid tumors, enabling full resection of the neoplasia, with acceptable morbidity. Nonetheless, its use when dissecting the neck and the facial nerve may result in cosmetic sequelae and unpleasant functional complications for some patients. Temporary changes to facial movement may happen in between 10% and 70% of the cases, and the rates of definitive paralysis of facial nerve branches vary^8^. Therefore, proper exposure in the region, associated with surgical expertise is fundamental to minimize these complications. The cervicomastoidfacial incision, described by Blair, in 1912 and modified by Bailey in 1941, has been established as the most utilized approach, since its bayonet shape extending from the pre-auricular region to the submandibular cervical region, provides a proper exposure of the entire parotid, associated with good functional results^9^, ^10^.

Despite all of this, the search for minimally invasive approaches and/or minimally perceptive scars has led a growing number of researchers to suggest that the modified rhytidectomy incision is a better alternative to the CMFI when one considers the cosmetic aspect of the scar. In an attempt to validate MR indications, we found that it was mainly indicated for females (67.9%) and young people, with a mean age lower than 35 years, which was also reported in a retrospective study carried out by Lohuis et al.^11^, in which 30 patients were submitted to parotidectomy through the MR approach and had mean age of 28.8 years. This suggests that the choice of MR for younger patients is due to the better cosmetic aspect of the incision, usually more important for this group of patients.

It is interesting to notice that, in a study assessing patient satisfaction with the scar resulting from the parotidectomy, no differences were seen between those operated by the MR or the CMFI approaches^6^. This result may be due to the large age difference between the two groups assessed by these researchers, with elder patients and in a higher number in the group operated by the cervicomastoidfacial approach (n = 59) compared to the MR (n = 20), because the cosmetic values may not be the same among different age ranges. Thus, as it happened in other studies^9^, ^11^, we found that the mean size of the resected tumor was similar in both groups (2.34 cm in MR versus 2.58 in CMFI), showing that the choice of incision is not limited by the exposure obtained by these different approaches. Amin et al.^12^ also showed the MR's versatility for total or partial parotidectomy regardless of tumor size, which in their data varied between 0.5 cm and 6.1 cm for CMFI.

Another frequently assessed aspect in the comparison of different approaches for the parotid is the relationship between the type of incision and the risk of facial motor dysfunction. In studies carried out by Lin and Lee^13^, ^14^ they did not observe differences between the incidence of facial paralysis in patients submitted to parotidectomy by the modified rhytidectomy incision or the classic cervicofacial approach, contrary to what was observed in our assessment, which associated the MR with facial movement functional results - which were significantly better than the CMFI, in the immediate post-operative (*p* = 0.0001), compared to the late one (*p* = 0.0146), although adjustment by age in the multivariate analysis suggested that late facial dysfunction is not directly associated to the type of incision made.

The lower incidence of facial motor dysfunction in patients submitted to parotidectomy through the MR approach was also noticed by Wasson et al.^6^, who found 34% of facial motor dysfunction in patients operated via the CMFI, compared to 20% in the cases approached the same way. The same occurrence of immediate facial motor dysfunction, regardless of patient age, may be associated with numerous factors, such as a limited dissection of the platysma muscle without the neck extension of the classical incision and a lower volume of the resected parotid. Although noticing that the total volume of the resected parotid was significantly lower than that of the cases approached by MR, we did not have incomplete resections or compromised margins in this group.

Cadaveric dissection studies comparing the MR surgical exposure to that obtained by the CMFI showed that both approaches have similar exposures - as per previously suggested by clinical trials carried out in patients operated for benign neoplasias^5^, ^15^, ^16^. Moreover, the MR was comparatively similar to the CMFI as to hospital stay duration and neck drainage volume, as observed in other comparative studies. We noticed that there was a greater incidence of salivary fistulas in those patients operated by the MR approach (13.3%) when compared to the classical approach (6.7%).

Nevertheless, such difference did not prove to be statistically significant, as it was also reported by Lee et al.^14^, although such approach sometimes requires a greater dissection of subcutaneous tissue for a proper exposure of the operatory field. MR has so far proved to be a proper access option for parotid benign tumors, for it yields a cosmetically more acceptable scar for most patients and functional results which are comparable to the classical approach. Nevertheless, one must stress that it has not been recommended to approach malignant neoplasias^17^ and, another not-so-well-known aspect about MR for parotidectomy is its association with the relative contraindications of such approach for cosmetic rhytidectomy, such as smoking, which may increase the likelihood of a tissue necrosis in 3 fold when compared to non-smokers^18^. It would be also adequate to remember the recommendation that such approach must be used by surgeons with experience in parotid surgery, and the classic approach is adequate for individuals under training and those with fewer surgical cases^11^.

## CONCLUSION

The modified rhytidectomy incision was mainly indicated for young and female individuals, it is associated with a lower incidence of facial movement temporary dysfunction. Further studies with more homogeneous and larger samples may establish the benefits of such approach for other groups of patients.
